# Preserved Morphology and Physiology of Excitatory Synapses in Profilin1-Deficient Mice

**DOI:** 10.1371/journal.pone.0030068

**Published:** 2012-01-11

**Authors:** Andreas Görlich, Anika-Maria Zimmermann, Doreen Schober, Ralph T. Böttcher, Marco Sassoè-Pognetto, Eckhard Friauf, Walter Witke, Marco B. Rust

**Affiliations:** 1 Neurobiology/Neurophysiology Group, University of Kaiserslautern, Kaiserslautern, Germany; 2 Max Planck Institute of Biochemistry, Martinsried, Germany; 3 Department of Anatomy, Pharmacology and Forensic Medicine and National Institute of Neuroscience-Italy, University of Turin, Turin, Italy; 4 Animal Physiology Group, University of Kaiserslautern, Kaiserslautern, Germany; 5 Mouse Biology Unit, European Molecular Biology Laboratory, Monterotondo, Italy; 6 Institute of Genetics, University of Bonn, Bonn, Germany; The Research Center of Neurobiology-Neurophysiology of Marseille, France

## Abstract

Profilins are important regulators of actin dynamics and have been implicated in activity-dependent morphological changes of dendritic spines and synaptic plasticity. Recently, defective presynaptic excitability and neurotransmitter release of glutamatergic synapses were described for profilin2-deficient mice. Both dendritic spine morphology and synaptic plasticity were fully preserved in these mutants, bringing forward the hypothesis that profilin1 is mainly involved in postsynaptic mechanisms, complementary to the presynaptic role of profilin2. To test the hypothesis and to elucidate the synaptic function of profilin1, we here specifically deleted profilin1 in neurons of the adult forebrain by using conditional knockout mice on a CaMKII-cre-expressing background. Analysis of Golgi-stained hippocampal pyramidal cells and electron micrographs from the CA1 *stratum radiatum* revealed normal synapse density, spine morphology, and synapse ultrastructure in the absence of profilin1. Moreover, electrophysiological recordings showed that basal synaptic transmission, presynaptic physiology, as well as postsynaptic plasticity were unchanged in profilin1 mutants. Hence, loss of profilin1 had no adverse effects on the morphology and function of excitatory synapses. Our data are in agreement with two different scenarios: i) profilins are not relevant for actin regulation in postsynaptic structures, activity-dependent morphological changes of dendritic spines, and synaptic plasticity or ii) profilin1 and profilin2 have overlapping functions particularly in the postsynaptic compartment. Future analysis of double mutant mice will ultimately unravel whether profilins are relevant for dendritic spine morphology and synaptic plasticity.

## Introduction

Dendritic spines are highly dynamic protrusions that form the postsynaptic part of most excitatory synapses. Changes in spine number and shape influence the strength of excitatory synaptic transmission and are thought to be the basis for learning and memory [1]–[3]. Actin is highly enriched in dendritic spines and is essential for their morphological changes (reviews: [4]–[5]). In fact, actin dynamics appear to be crucially important for structural adaptations of neuronal circuits associated with learning and memory formation [6]–[10]. Hence, it is important to understand the detailed mechanisms that link actin dynamics and synaptic plasticity.

Actin dynamics critically depend on the activity of profilins that make actin monomers available for the incorporation into actin filaments and direct them to the site of actin polymerization [11]. Of the four identified profilin isoforms, only profilin1 and profilin2 are expressed in the mouse central nervous system [12]. Both proteins are located in synaptic structures [13] and show an activity-dependent recruitment to dendritic spines in neuronal cultures [14]–[15]. Moreover, analysis of organotypic hippocampal cultures suggests a role for profilin1 and profilin2 in dendritic spine morphology [16]. Based on these studies, it was proposed that profilins have an important role in activity-driven actin dynamics in dendritic spines and synaptic plasticity [5]. Accordingly, a learning-dependent recruitment of profilins into dendritic spines was observed in fear-conditioned rats [17]. However, these data are difficult to reconcile with the phenotype of profilin2-deficient mice as these mutants display normal synaptic plasticity, learning, and memory [13]. Instead, they show increased neurotransmitter release, pointing to a critical role of profilin2 in presynaptic excitability. Therefore, our aim was to investigate the discrepancy of *in vitro* and *in vivo* findings and, for the first time, to elucidate the isoform-specific synaptic function of profilin1 *in vivo*.

Profilin1-deficient embryos die during early development [18] and profilin1 inactivation during brain development interferes with neuronal migration and brain development [19]. Thus, analysis of profilin1 in synaptic plasticity requires the deletion of profilin1 specifically in the adult forebrain. To do so, we crossed conditional profilin1 mutants (Pfn1^flx/flx^) with a transgenic line expressing cre under the control of the Ca^2+^/calmodulin-dependent protein kinase II α subunit (CaMKII-cre) [20]–[21]. Our analysis revealed normal synapse density in profilin1 mutant mice and virtually no defect in synapse morphology, with the exception of a slight increase in the neck length of mushroom-like spines. Moreover, basal synaptic transmission, presynaptic physiology, as well as postsynaptic plasticity were independent of profilin1 activity. Hence, our data demonstrate that profilin1 inactivation has no adverse effects on excitatory synapses. We suggest that profilin1 and profilin2 have the capacity to compensate each other in postsynaptic structures. Analyses of double mutant mice are required to ultimately unravel the postsynaptic function of profilins *in vivo*.

## Materials and Methods

### Ethics Statement

Treatment of mice was in accordance with the German law for conducting animal experiments and followed the NIH guide for the care and use of laboratory animals. Killing of mice for tissue analysis was approved by the Landesuntersuchungsamt Rheinland-Pfalz (23 177-07/G09-2-001), mouse husbandry and breeding was approved by the City of Kaiserslautern – Referat Umweltschutz.

### Animals

Forebrain-specific deletion of profilin1 was achieved by crossing the conditional profilin1 allele (Pfn1^flx/flx^) [20] with a transgenic cre-expressing line, driven by Ca^2+^/calmodulin-depedent protein kinase II α subunit (CaMKII-cre) [21].

### Biochemistry

#### Protein lysates

Brain extracts were prepared by homogenizing fresh tissue in ice-cold lysis buffer (in mM): 20 Tris-HCl (pH 8.0), 100 NaCl, 5 EGTA, 2 EDTA, supplemented with 0.5% TritonX-100 and EDTA-free complete protease inhibitor mix (Roche) using a tightly fitting douncer.

*Preparation of hippocampal synaptosomes* was essentially performed as described before [9]. Briefly, tissue was homogenized in homogenization solution containing (pH 7.4, in mM): 320 sucrose, 1 EDTA, 5 HEPES, supplemented with 0.1% bovine serum albumin and EDTA-free Complete protease inhibitor mix (Roche) using a tight fitting douncer. After removing nuclei and cell debris, material containing synaptosomes was resuspended in Krebs-Ringer solution (pH 7.4) containing (in mM): 140 NaCl, 5 KCl, 1 EDTA, 10 HEPES, 5 glucose. Synaptosomes were enriched on a floatation gradient consisting of 35% Percoll. Anti-β tubulin antibody was purchased from Sigma-Aldrich (clone TUB 2.1, #T5201; 1∶5,000). Antibodies that specifically recognize profilin1 and profilin2 were used as described before [12]–[13].

### Electrophysiology

#### Tissue preparation

4–6 week-old mice were sacrificed by cervical dislocation, and their brains were rapidly removed and dissected in chilled solution (4°C) containing (in mM): 87 NaCl, 2 KCl, 0.5 CaCl_2_, 7 MgCl_2_, 26 NaHCO_3_, 1.25 NaH_2_PO_4_, 25 glucose, 75 sucrose, bubbled with a mixture of 95% O_2_/5% CO_2_, leading to a pH of 7.4. 300–370 µm-thick horizontal hippocampal slices were cut with a VT1200S vibratome (Leica), preincubated for 30 min at 37°C, and then transferred to recording solution (room temperature) containing in (mM): 125 NaCl, 2.5 KCl, 2 CaCl_2_, 1.3 MgSO_4_, 26 NaHCO_3_, 1.25 NaH_2_PO_4_, 10 glucose, 2 sodium pyruvate, 3 myo-inositol, 0.44 ascorbic acid, bubbled with a mixture of 95% O_2_/5% CO_2_, leading to a pH of 7.4. Slices rested in this solution for at least one hour before recordings began.

#### Single cell recordings

Patch pipettes had resistances of 4–8 MΩ when filled with a solution containing (in mM): 117.5 CsMeSO_4_, 2.5 CsCl, 8 NaCl, 10 HEPES, 10 TEA, 0.2 EGTA, 4 Na_2_ATP, 0.6 Na_2_GTP, 5 QX-314 pH was adjusted to 7.2 with CsOH. Slices were transferred into the recording chamber, which was continuously perfused at a rate of 1.5–2 ml/min with recording solution at room temperature. CA1 hippocampal neurons were visualized with DIC-infrared optics using a 60×/1.0 water immersion objective on an upright Eclipe E600-FN microscope (Nikon). Electrophysiological responses were recorded with an EPC 10 patch-clamp amplifier and PatchMaster and FitMaster software (HEKA Elektronik). For measurements of miniature EPSCs (mEPSCs), the bath solution contained 4 mM CaCl_2_ and 4 mM MgSO_4_. During recordings, 0.5 µM tetrodotoxin (TTX; Ascent Scientific), 100 µM picrotoxin (Ascent Scientific), and 250 µM trichlormethiazide (TCM; Sigma-Aldrich) were added to the recording solution. CA1 hippocampal neurons were voltage clamped at −70 mV, and spontaneous mEPSCs were recorded for five minutes. Amplitude and inter-event intervals (IEI) were analyzed with miniAnalysis (Synaptosoft), with an amplitude threshold of 3.5 pA.

#### Field potential recordings

For stimulation of Schaffer collaterals, monopolar glass electrodes, filled with recording solution, were placed in the *stratum radiatum* of the CA1 region. For field potential experiments, pipettes were filled with 3 M NaCl, and fEPSPs were measured at a stimulus intensity that elicited amplitudes that were ∼30–50% of the maximum. Input-output curves were built by measuring the fiber volley and fEPSP responses evoked by stimulating afferent fibers with current intensities ranging from 20 to 300 µA. Paired-pulse ratio (PPR) was analyzed by applying pairs of stimuli at the following inter-stimulus intervals (ISI; in ms): 10, 15, 25, 50, 75, 100, 150, 200. LTP was elicited either by a single one-second 100 Hz train or by 10 bursts of four pulses at 100 Hz, separated by 200 ms (theta-burst stimulation). For the measurement of long-term depression (LTD) experiments, mice were in the age of postnatal day 17 (P17) to P21. For the induction of LTD, a low-frequency stimulation (LFS) was used, consisting of 900 pairs of stimuli (distance 50 ms) at 1 Hz.

### Morphology

#### Golgi staining

Mice aged 10–12 weeks were used. The FD Rapid GolgiStain™ kit (FD Neurotechniques) was used for Golgi staining; tissue impregnation and tissue section staining were performed according to the manufacturer's data sheet. Briefly, mice were perfused with 4% formaldehyde and brains were quickly removed from the skull and postfixed in the same fixative overnight. After incubation in impregnation solution and solution C, brains were imbedded in gelatin-albumin and cut into 100 µm coronal sections using a vibrating microtome (Campden Instruments Ltd.). Sections were mounted on gelatinized glass slides, further processed for the Golgi staining procedure, and finally mounted in Entellan (Merck). High magnification images of 2^nd^ order dendritic branches in the hippocampal CA1 *stratum radiatum* were generated with an Axioskop microscope and a Plan-Neofluar 100×/1.30 oil immersion objective (Carl Zeiss). Spine density and morphology were measured using ImageJ 1.42q imaging software (NIH). Image acquisition and morphometric analyses were performed by an experimenter blind to the genotype of the mice. *Electron microscopy:* 10–12 week-old mice were perfused with 1% formaldehyde/1% glutaraldehyde in phosphate buffer (0.1 M PB, pH 7.4). Their brains were postfixed in the same fixative overnight, and small specimens taken from the dorsal hippocampus were postfixed in 1% OsO4 in 0.1 M cacodylate buffer, dehydrated, and embedded in epoxy resin. Ultrathin sections were stained with uranyl acetate and lead citrate and observed in a JEM-1010 transmission electron microscope (Jeol) equipped with a side-mounted CCD camera (Mega View III, Soft Imaging System). Spine density was assessed by analyzing 192 digitized images from four mice of each group. Images (30,000× magnification, area size 14.66 µm^2^) were captured in the proximal part of CA1 *stratum radiatum*. Morphometric analysis was done on electron micrographs taken at 75,000× using ImageJ 1.42q imaging software. Synaptic structures were identified by presynaptic terminals with at least three synaptic vesicles, a visible synaptic cleft and a well-defined postsynaptic density. Image acquisition and morphometric analysis were performed by an experimenter blind to the genotype of the mice.

### Statistical analysis

The unpaired two-tailed *t*-Student's test was used for statistical analysis.

## Results

### Forebrain-specific deletion of profilin1 and unaltered profilin2 expression levels

**Figure 1:**
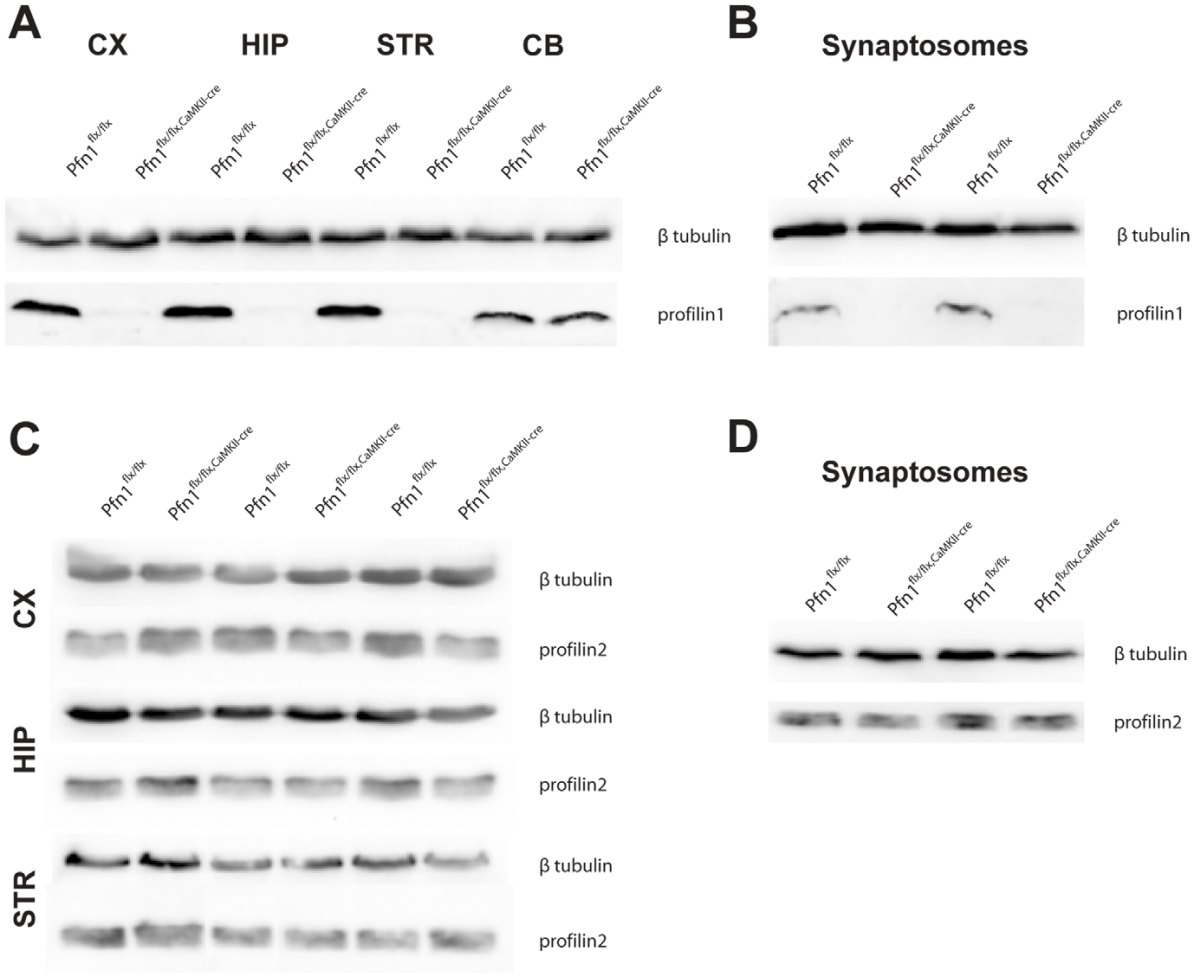
Deletion of profilin1 in Pfn1^flx/flx,CaMKII-cre^ mice. (**A**) Immunoblot analysis in different brain regions from an adult Pfn1^flx/flx^ control and an adult Pfn1^flx/flx,CaMKII-cre^ mutant (P70), revealing efficient deletion of profilin1 in the forebrain of mutants. In all three forebrain tissues (cortex (CX), striatum (STR), hippocampus (HIP)), profilin1 expression was almost undetectable in mutants. In contrast, profilin1 expression level was unchanged in the cerebellum (CB), in which cre is not expressed. Identical results were obtained when investigating profilin1 expression levels in two other Pfn1^flx/flx,CaMKII-cre^ mice. (**B**) Immunoblot analysis of hippocampal synaptosomes, demonstrating the absence of profilin1 from synaptic structures in mutants. (**C**) No changes in profilin2 expression were found in the cortex, hippocampus, or striatum of three individual profilin1-deficient mice. (**D**) Normal profilin2 content in hippocampal synaptosomes from two individual Pfn1^flx/flx,CaMKII-cre^ mice. Expression of β tubulin was examined to control protein load in A–D.

To investigate the role of profilin1 in synapse physiology and plasticity, we generated conditional mutants with a selective deletion of the profilin1 gene in principal neurons of the adult forebrain (Pfn1^flx/flx,CaMKII-cre^). Immunoblot analysis of protein lysates from various brain regions of Pfn1^flx/flx,CaMKII-cre^ mice at P70 confirmed the efficient deletion of profilin1 in all three forebrain structures examined (cortex, hippocampus, striatum; Fig. 1A). As expected, no changes in profilin1 expression levels were detectable in lysates from the cerebellum, where cre is not expressed [21]. Notably, no profilin1 immunoreactivity was detectable in hippocampal synaptosomes of Pfn1^flx/flx,CaMKII-cre^ mice, demonstrating the absence of any profilin1 from synaptic structures (Fig. 1B). In none of the three Pfn1^flx/flx,CaMKII-cre^ forebrain regions (Fig. 1C), nor in hippocampal synaptosomes (Fig. 1D), did we find evidence for different expression levels of profilin2.

### Spine density and synapse ultrastructure are normal in Pfn1^flx/flx,CaMKII-cre^ mice

**Figure 2:**
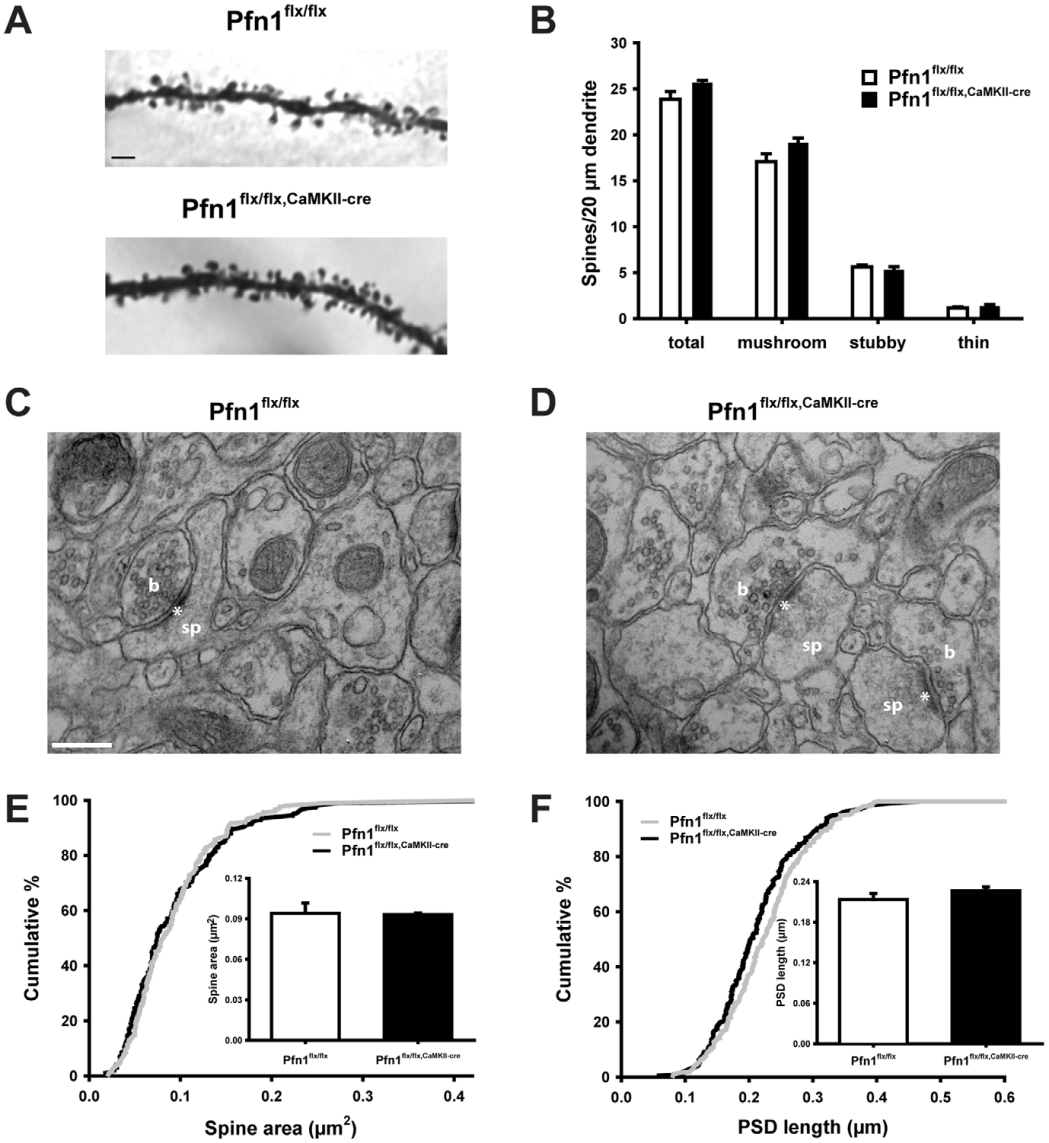
Unaltered spine density and morphology in hippocampal CA1 region of Pfn1^flx/flx,CaMKII-cre^ mice. (**A**) Representative images of 2^nd^ order dendritic branches of Golgi-stained pyramidal cells in the hippocampal CA1 *stratum radiatum*. Scale bar: 2 µm. (**B**) Unaltered spine density in Pfn1^flx/flx,CaMKII-cre^ mice. Spines were morphologically categorized into mushroom-like, stubby, and thin spines (>1,000 µm length of dendritic branches for both groups, four mice per group). Representative electron micrographs of CA1 *stratum radiatum* of (**C**) Pfn1^flx/flx^ controls and (**D**) Pfn1^flx/flx,CaMKII-cre^ mice. Scale bar in C: 200 nm. b: presynaptic bouton, sp: dendritic spine, *: postsynaptic density. Unaltered spine area (**E**) and PSD length (**F**) in Pfn1^flx/flx,CaMKII-cre^ mice as deduced from cumulative distributions and mean values (insets in E and F).

The activity-dependent recruitment to dendritic spines in dissociated hippocampal neurons suggests that profilin1 is important for the morphology of postsynaptic compartments [15]. To address this point, we visualized dendritic spines in Golgi-stained neurons from coronal sections of control and Pfn1^flx/flx,CaMKII-cre^ mice at P70–P80. For the analysis of dendritic spine density and morphology, we chose 2^nd^ order dendritic branches in the CA1 *stratum radiatum* (Fig. 2A), in which profilin1 immunoreactivity is reportedly particularly pronounced [15]. The density of spines was similar in controls (23.9±0.9 spines/20 µm dendrite) and Pfn1^flx/flx,CaMKII-cre^ mice (25.6±0.3 spines/20 µm dendrite; Fig. 2B). Moreover, there was no difference in the density of mushroom-like, stubby, or thin spines between the two groups. Likewise, morphometric analyses revealed virtually no difference in spine morphology between controls and mutants, except for a slight increase in the neck length of mushroom-like spines (controls: 0.46±0.02 µm, mutants: 0.52±0.01; P<0.05; Table 1). Electron microscopic analysis in CA1 *stratum radiatum* (Fig. 2C–D) confirmed that the density of excitatory synapses was unchanged in the absence of profilin1 (controls: 4.71±0.46 synapses/10 µm^2^, n = 3386 µm^2^ from four mice; mutants: 4.66±0.35 synapses/10 µm^2^, n = 2829 µm^2^/4 mice). Moreover, spine area and length of the postsynaptic density (PSD) were indistinguishable between controls and mutants (Fig. 2E–F).

**Table 1:** Dendritic spine morphology.

		Pfn1^flx/flx^		Pfn1^flx/flx,CaMKII-cre^
**mushroom-like**	**head perimeter (µm)**	2.31±0.08		2.21±0.09
	**neck length (µm)**	0.46±0.02	*	0.52±0.01
**Stubby**	**perimeter (µm)**	2.53±0.05		2.46±0.04
**Thin**	**length (µm)**	1.19±0.05		1.16±0.05

Shown are the mean values (±SEM).

* :P<0.05.

Profilin2 has been implicated in the organization of synaptic vesicles [13], and we wanted to know whether profilin1 fulfills a similar function. We therefore analyzed the synaptic vesicle organization in Pfn1^flx/flx,CaMKII-cre^ mice and found no changes in the vesicle density (control: 178.99±8.25 vesicles/µm^2^, n = 182 presynaptic terminals/4 mice; mutant: 160.44±8.75 vesicles/µm^2^, n = 177 presynaptic terminals/4 mice) or in the density of docked vesicles (control: 12.92±1.02 docked vesicles/µm of active zone, n = 129 presynaptic terminals/4 mice; mutant: 12.56±0.66 docked vesicles/µm, n = 141 presynaptic terminals/4 mice). In summary, synapse density, spine morphology, as well as synaptic vesicle density and organization were all unchanged in Pfn1^flx/flx,CaMKII-cre^ mice.

### Profilin1 is dispensable for pre- and postsynaptic physiology

**Figure 3:**
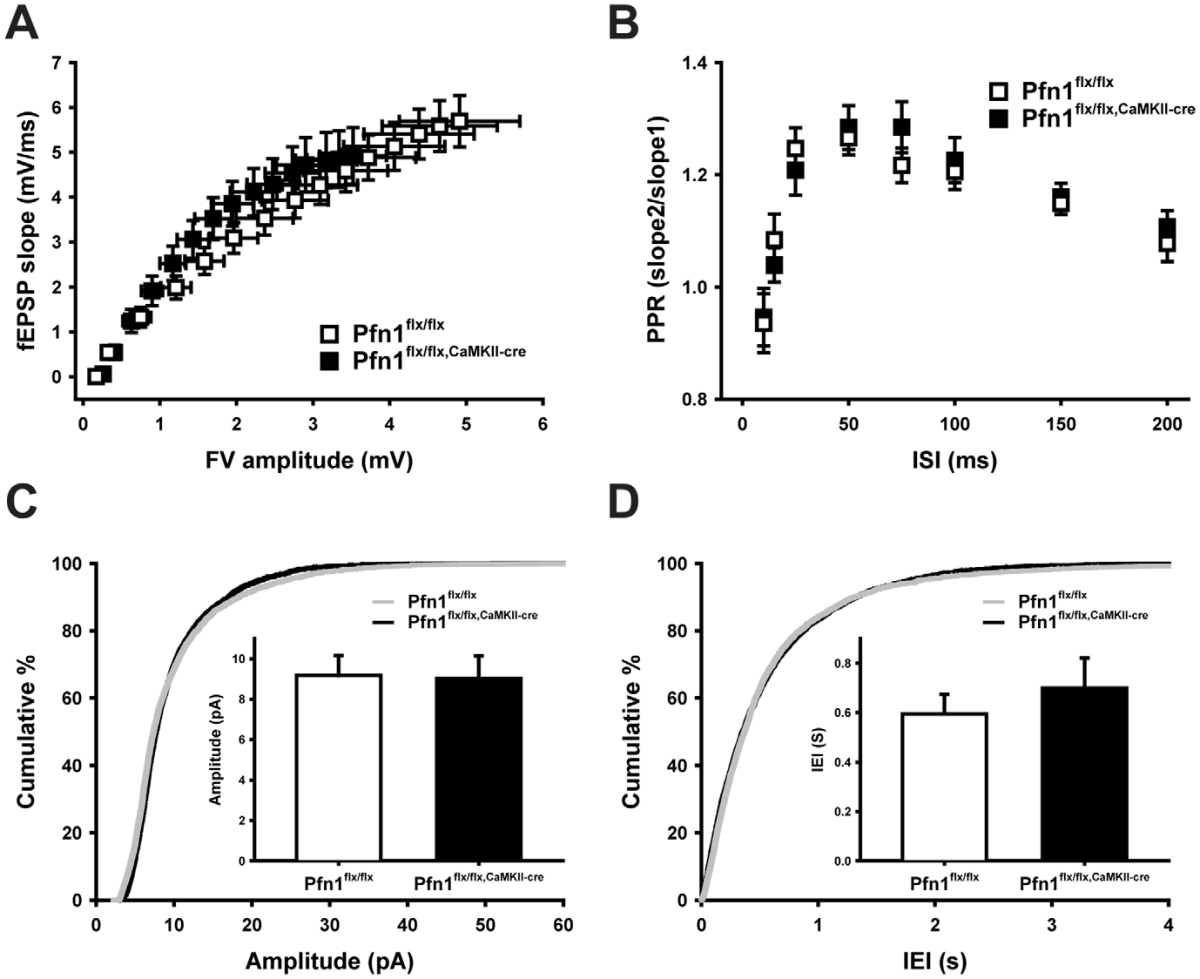
Normal presynaptic function of hippocampal CA3-CA1 synapses in Pfn1^flx/flx,CaMKII-cre^ mice. (**A**) Basal synaptic transmission, as deduced from input-output curves, was normal in Schaffer-collateral-CA1 synapses of Pfn1^flx/flx,CaMKII-cre^ mice (n = 15 for controls and 10 for mutants). (**B**) In Pfn1^flx/flx,CaMKII-cre^ mice, no changes were found in paired-pulse ratios (PPR; n = 14 for controls, n = 17 for mutants) at various inter-stimulus intervals (ISI; 10–200 ms). Cumulative curves of amplitudes (**C**) and inter-event intervals (IEI) of mEPSCs (**D**) were virtually equal between genotypes (n = 8 in each group). Insets in C and D depict mean values.

Profilin2 is required for presynaptic function, yet not for postsynaptic plasticity [13]. We next set out to test whether profilin1 plays a similar or complementary role in pre- and postsynaptic physiology. To do so, we first assessed whether general synaptic transmission and synaptic efficiency were affected in the absence of profilin1. We recorded extracellular field potentials in the CA1 region in acute hippocampal slices upon stimulation of the Schaffer collateral pathway with intensities ranging from 20–300 µA. The resulting input-output curves revealed no differences in presynaptic fiber volley amplitude or postsynaptic fEPSP slope between the genotypes (Fig. 3A). To elucidate a potential involvement of profilin1 in presynaptic physiology, we determined the paired-pulse ratio (PPR) at various inter-stimulus intervals (ISI; 10–200 ms) and again found no differences between Pfn1^flx/flx,CaMKII-cre^ mice and controls (Fig. 3B). Moreover, the amplitudes and the inter-event intervals (IEI) of miniature excitatory postsynaptic currents (mEPSCs) obtained from patch-clamped CA1 pyramidal neurons were not changed in profilin1-deficient mice (Fig. 3C–D). Together our data demonstrate that presynaptic vesicle loading, vesicle release probability, and the vesicle release machinery are not altered in Pfn1^flx/flx,CaMKII-cre^ mice.

**Figure 4:**
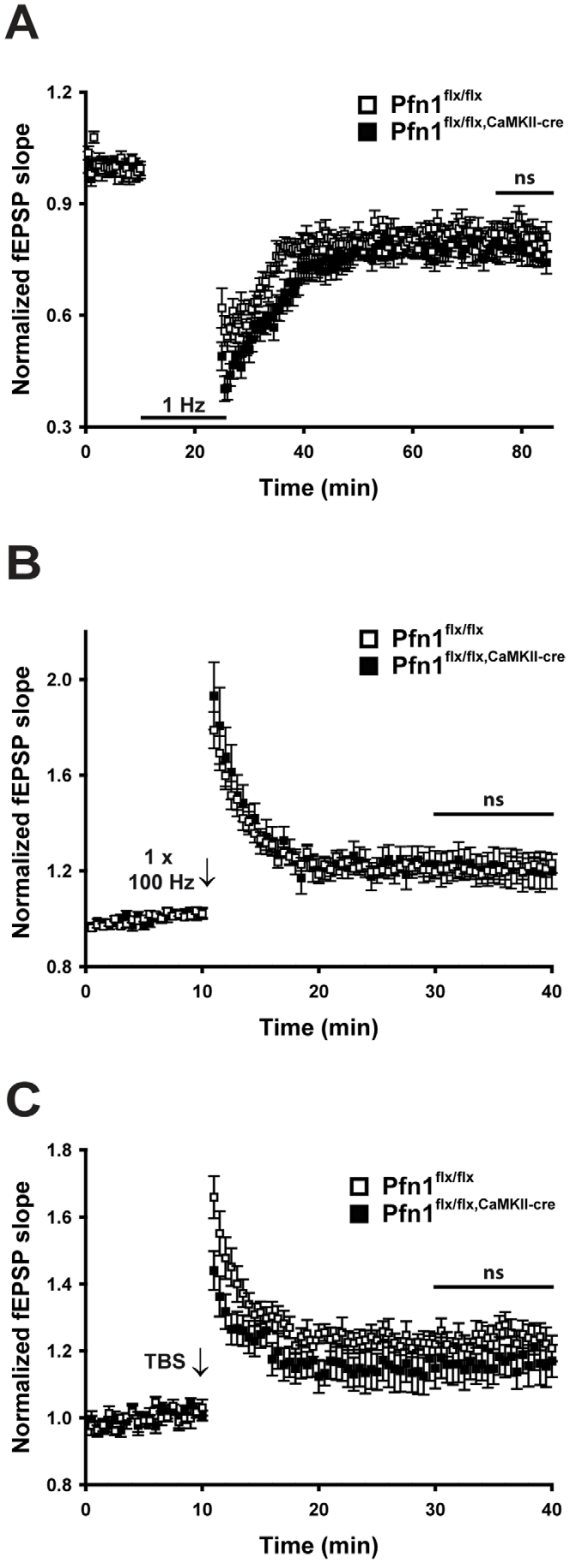
Unimpaired synaptic plasticity in the absence of profilin1. (**A**) In Pfn1^flx/flx,CaMKII-cre^ mice, no difference was found in LTD induced by low frequency stimulation (1 Hz) of 15 min duration (n = 9 for controls, n = 12 for mutants) when analyzing the last 10 min of the recordings. LTP induced by either a single 100 Hz tetanus of 1 s duration (**B**) or by theta-burst stimulation (TBS) (**C**) was also not different between genotypes (1×100 Hz: n = 10 for controls and 8 for mutants; TBS: n = 10 for both groups). ns: not significant.

Localization experiments have suggested a potential role of profilin1 in postsynaptic physiology [15]. We tested this hypothesis in our genetic model by measuring synaptic strength modulation during LTD and LTP. When LTD was evoked through paired stimulation at 1 Hz for 15 min, we did not see a significant difference in the induced steady state (45–85 min of the recording) between controls and profilin1 mutants (Fig. 4A; P>0.05, considering the last 10 min of the recordings for statistical analysis). Also, when we induced LTP by a single 100 Hz tetanus of 1 s duration (1×100 Hz) or by theta-burst stimulation (TBS), we did not find any significant differences in the resulting steady states (25–40 min of the recording) between controls and profilin1 mutants (Fig. 4B–C; P>0.05 in both experiments considering the last 10 min of the recordings for statistical analysis). Hence, our data demonstrate unchanged synaptic plasticity of hippocampal CA3-CA1 synapses in the absence of profilin1.

## Discussion

A depolarization- and NMDAR-driven recruitment of profilin1 and profilin2 to postsynaptic sites of excitatory synapses has been demonstrated in studies on dissociated hippocampal neurons [14]–[15]. Accordingly, analysis of organotypic hippocampal cultures suggests a role of profilin1 and profilin2 in dendritic spine morphology [16]. Based on these experiments, it was suggested that profilins are involved in actin turnover in postsynaptic compartments, in activity-dependent morphological changes of dendritic spines, and in postsynaptic plasticity [4]–[5], [22]. These ideas were supported by results obtained from fear-conditioned rats which showed a learning-induced translocation of profilins into dendritic spines of lateral amygdala neurons [17]. However, as the antibody used in this study recognizes both profilin isoforms, the relative contribution of profilin1 and profilin2 to postsynaptic mechanisms remained unclear. For example, profilin2 is present in a much larger fraction of dendritic spines than profilin1 [15]. Thus, a predominant contribution of profilin2 to postsynaptic plasticity was postulated [4], [23]–[24], which, however, could not be confirmed *in vivo* in profilin2-mutant mice [13]. Moreover, various forms of synaptic plasticity (LTP, LTD), as well as learning and memory, were normal in this mouse model [13]. Thus, the discrepancy between the results obtained from *in vitro* experiments and those of profilin2-mutant mice raised two important questions: First, do profilins indeed play a role in dendritic spines *in vivo*? Second, which profilin isoform does then contribute to postsynaptic mechanisms?

To address these questions, we chose a genetic approach: we deleted profilin1 specifically in principal neurons of the mouse forebrain by using a conditional knockout mouse model and a transgene expressing cre under the control of the CaMKII-α [20]–[21]. As profilin1 expression levels are particularly high in hippocampal neurons [15], we chose CA1 hippocampal pyramidal cells for morphometric analysis and hippocampal CA3-CA1 projections for the characterization of profilin1 function in excitatory synapses.

By two independent approaches (Golgi-staining and electron microscopy), we found that inactivation of profilin1 has no effect on the organization of synaptic vesicles or on the density and morphology of excitatory synapses, except for a slight increase in the neck length of mushroom-like spines. Moreover, our extensive electrophysiological analyses revealed that basal synaptic transmission, presynaptic mechanisms (vesicle loading, vesicle release probability), and postsynaptic plasticity (LTP, LTD) are fully preserved in the absence of profilin1. Together, these data indicate that inactivation of profilin1 has no adverse effects on the structure and function of excitatory synapses. Thus, in contrast to previous suggestions, we show that profilin1 is not essential for dendritic spine morphology and synaptic plasticity.

Our data are in agreement with two possible scenarios. First: profilins are not relevant for actin regulation in postsynaptic structures, activity-dependent morphological changes of dendritic spines, and synaptic plasticity. Second: profilin1 and profilin2 have the capacity to compensate each other in postsynaptic structures. In agreement with the latter suggestion, down-regulation of profilin2 is functionally compensated by profilin1, specifically in dendritic spines [16]. Whether this also occurs *in vivo* still needs to be addressed experimentally. Future analyses of double-mutant mice are therefore needed for a comprehensive understanding of profilin function in synaptic physiology and will ultimately unravel whether profilin activity is relevant for dendritic spine morphology and postsynaptic plasticity.
